# Tonsillar Leishmaniasis: A Rare Clinical Entity Mimicking Malignancy in the Oropharynx – A Case Series from Northeastern Italy

**DOI:** 10.1007/s12105-025-01773-3

**Published:** 2025-03-26

**Authors:** Giulia Querzoli, Margherita Ortalli, Stefania Varani, Matteo Errani, Andrea Ambrosini-Spaltro, Marina Del Vecchio, Anna Rita Lombardi, Paolo Rinaldi, Carlo Biagetti, Riccardo Albertini, Luca Amorosa, Alessandro Rosti, Marco Trebbi, Paolo Farneti, Ernesto Pasquini, Antonio Mastroianni, Maria Pia Foschini, Valeria Gaspari

**Affiliations:** 1 Pathology Unit, IRCCS Azienda Ospedaliero Universitaria di Bologna, Bologna, Italy; 2 Department of Medical and Surgical Sciences (DIMEC), University of Bologna, Via Albertoni 15, 40100 Bologna, Italy; 3 Unit of Microbiology, IRCCS Azienda Ospedaliero-Universitaria di Bologna, Bologna, Italy; 4 School of Anatomic Pathology, Department of Biomedical and Neuromotor Sciences, University of Bologna, 40126 Bologna, Italy; 5 Pathology Unit, Morgagni-Pierantoni Hospital, Forlì, Romagna, AUSL Italy; 6 Pathology Unit, Degli Infermi Hospital, Rimini, Romagna, AUSL Italy; 7 UOC Malattie Infettive Ospedale degli Infermi Rimini AUSL Romagna, Romagna, Italy; 8 Otorhinolaryngology Unit, Maggiore Hospital, AUSL Bologna, Bologna, Italy; 9 Department of Otolaryngology - Head and Neck Surgery, IRCCS Azienda Ospedaliero- Universitaria di Bologna, Bologna, Italy; 10 UOC ORL, Ospedale degli Infermi Rimini, Dipartimento Chirurgico, AUSL, Romagna, Italy; 11 ENT Unit, Bellaria Hospital, AUSL Bologna, 40139 Bologna, Italy; 12 Infectious and Tropical Diseases Unit, Department of Specialty Medicine, “Annunziata” Hub Hospital, Azienda Ospedaliera di Cosenza, Cosenza, Italy; 13 Department of Biomedical and Neuromotor Sciences (DIBINEM), Alma Mater Studiorum, Unit of Anatomic Pathology, University of Bologna, Bellaria Hospital, Bologna, Italy; 14 Dermatology Unit, IRCCS Azienda Ospedaliero-Universitaria di Bologna, Policlinico S. Orsola Malpighi, 40126 Bologna, Italy

**Keywords:** Tegumentary leishmaniasis, Granulomatous lesions, Endemic regions, Tonsillar leishmaniasis, Oropharyngeal malignancies

## Abstract

**Purpose:**

Tegumentary leishmaniasis (TL) is a neglected disease in Europe, often underdiagnosed or misdiagnosed due to its variable clinical presentation. Mucosal leishmaniasis (ML) is a rare manifestation of TL, and isolated tonsillar leishmaniasis is an even rarer finding, with very few reported cases. This study aims to expand knowledge on this unusual clinical entity by describing five cases of isolated tonsillar leishmaniasis diagnosed in the Emilia-Romagna region (ERR), northeastern Italy, emphasizing diagnostic challenges and treatment outcomes.

**Methods:**

Between January 2014 and December 2024, all consecutive patients presenting with unilateral tonsillar swelling and pharyngodynia were evaluated at otolaryngology units in ERR hospitals. Histopathological analysis, special stains (H&E, Giemsa, PAS, Ziehl-Neelsen), and immunostaining (CD1a) were performed at the referring hospital. Microbiological confirmation was obtained through real-time PCR targeting Leishmania kDNA and rRNA. Treatment was administered according to current TL guidelines.

**Results:**

We included five patients who presented with unilateral tonsillar swelling mimicking malignancy and with a histological diagnosis of non-necrotizing granulomas. Histology revealed amastigotes in four cases. PCR confirmed Leishmania infection in all cases. Treatment with liposomal amphotericin B or pentamidine led to complete clinical remission.

**Conclusion:**

Isolated tonsillar leishmaniasis should be considered in the differential diagnosis of head and neck tumors, especially in endemic regions. Histological and molecular tools are essential for accurate diagnosis. Increased awareness among clinicians and pathologists is necessary to improve recognition and management of this rare presentation.

**Supplementary Information:**

The online version contains supplementary material available at 10.1007/s12105-025-01773-3.

## Introduction

Leishmaniasis is a neglected tropical disease caused by protozoan pathogens transmitted to vertebrate hosts by phlebotomine sandflies [1]. Several rodent and canine species have been identified as reservoirs, with sandflies serving as vectors; rarely, transmission can occur through congenital means or via blood transfusions. The clinical presentation of leishmaniasis is influenced by a complex interplay between the virulence of the infecting *Leishmania* species and the host’s immune response, leading to a spectrum of disease manifestations, from localized skin lesions to widespread involvement of the reticuloendothelial system [2–3].

Leishmaniasis in humans is traditionally classified into three main clinical forms, including systemic and localized disease. Visceral leishmaniasis (VL) is a systemic condition, with an incubation period ranging from 2 to 6 months. It is characterized by recurrent fever, spleen enlargement, and anemia, and if left untreated, it can be life-threatening. Localized leishmaniasis includes cutaneous leishmaniasis (CL) and mucosal or mucocutaneous leishmaniasis (ML or MCL). CL has a shorter incubation period, typically between 2 weeks and 6 months. Although it is not fatal, it causes skin lesions that can sometimes be disfiguring. ML is a rare form, especially in Europe, that primarily affects the mucous membranes of the nose, mouth, and throat. Each form presents distinct clinical manifestations, with VL being the most dangerous, CL the most common, and ML/MCL the least frequent but still significant [1]. CL and ML are collectively referred to as tegumentary leishmaniasis (TL).

TL is a neglected disease in Europe: it is often misdiagnosed or diagnosed with delay, and its surveillance is not mandatory in many countries, such as Austria, Belgium, France and Germany [4]. In Italy, case notification for human leishmaniasis is mandatory, but a high rate of under notification is estimated as TL is often a benign disease that does not require hospitalization [5]. In 2018, the first report on the epidemiological situation of human leishmaniasis in Italy was published by the World Health Organization: between 1998 and 2016, only 800 cases of CL were notified all over the country [6]. Historically, CL cases occur in the Tyrrhenian coastline, southern peninsular regions, and the main islands [7]. Nevertheless, the epidemiological pattern of leishmaniasis in Italy is changing, leading to an increase in human cases in the northern part of the country. An upsurge of TL cases was recently observed in the Emilia-Romagna region (ERR) in northeastern Italy, with clusters in areas between the provinces of Modena and Bologna and in the provinces of Forlì-Cesena and Rimini [8]. TL is important as the disease is associated with physical deformities and psychological disorders, affecting the health and wellness of the patient [9].

Diagnosing TL accurately is challenging due to its wide range of clinical symptoms; however, proper TL diagnosis is crucial for ensuring effective treatment and preventing the infection’s spread and it relies on a combination of epidemiological history, clinical manifestations, and laboratory tests. The latter include histopathological examination of skin biopsies, as well as molecular tests for detecting *Leishmania* DNA [10].

ML is uncommon, but an exclusive tonsillar localization of *Leishmania* is an even rarer finding in the clinical routine. Only a few cases of tonsillar leishmaniasis were previously reported [3, 11–14] as summarized in Table 1. Given the limited data on this clinical entity, expanding knowledge is crucial to enabling physicians and pathologists to correctly recognize and diagnose it. Here, we describe five cases of isolated tonsillar leishmaniasis that were identified in ERR, northeastern Italy.

**Table 1:** Summary of reported cases of isolated tonsillar leishmaniasis in the literature

Reference	Sex/Age(yr)	Country of origin	Comorbidities	Previous surgery	Travel history	Animal
Laudadio, P. 1984 [11]	M/41	Italy	Arterial hypertension	NA	NA	NA
Cortés P, et al. 1997 [12]	M/30	Spain	HIV	Facial reconstructive surgery, due to osteochondritis of unknown etiology that had caused a saddle-nose deformity.	no	no
Aliaga L, et al. 2003 [3]	F/61	Spain	no	NA	no	NA
Kouyialis S.et al. 2005 [13]	M/34	Greece	NA	NA	NA	NA
Hoyos CL, et al. 2019 [14]	M/30	Argentina	NA	NA	NA	NA

NA not available

## Materials and Methods

Patients presented at the Units of Otolaryngology of the Bellaria and Maggiore Hospitals in Bologna, Morgagni-Pierantoni Hospital in Forlì, and Infermi Hospital in Rimini, Italy between January 2014 and December 2024.

### Pathological Examination

All the tissue samples were formalin fixed, and paraffin embedded (FFPE) at the Units of Anatomic Pathology of Bellaria Hospital in Bologna, Morgagni-Pierantoni Hospital in Forlì and Infermi Hospital in Rimini. Serial sections were obtained and stained with Hematoxylin-eosin (H&E), PAS and after diastase digestion, Ziehl-Neelsen and Giemsa. In some hospitals, additional immunostaining with CD1a antibody was performed. Staining was performed using the Ventana immunostainer, with the EP3622 Rabbit Monoclonal Antibody from Ventana-Roche Tissue Diagnostics.

### Microbiological Examination

Ten-micron thick sections were obtained from all cases and sent to the regional reference laboratory for human leishmaniasis at the Unit of Microbiology (University Hospital of Bologna) for *Leishmania* DNA search. DNA was extracted from FFPE sections and real-time PCR was carried out with two in-house assays, one targeting a segment of the small-subunit ribosomal RNA (rRNA) gene and the other targeting a region of minicircle kinetoplast (k)DNA as previously described [15].

### Treatment

Treatment was performed at the Unit of Dermatology at University Hospital of Bologna, at the Infectious Disease Unit at Morgagni-Pierantoni Hospital of Forlì and at Infermi Hospital, Rimini, according to the currently available guidelines for TL [16].

## Results

### Clinical Signs

Clinical data are summarized in Table 2. Details on each case are included as supplementary files.

**Table 2:** Clinical characteristics of patients with tonsillar leishmaniasis, Emilia-Romagna region, Northeastern Italy (2014–2024)

Patient	Sex	Age	Duration of symptoms (months)	Comorbidities	Therapy	Follow-up (months)
Case 1	F	53	1	Smoker; DM type II	Liposomial Amphotericin B 3 mg/kg/day at day 1–5, 10 and 21	1 after treatment: asymptomatic
Case 2	M	42	1	None	Pentamidine isethionate (4 mg/kg, three infusions over 5 days), plus Miltefosine (50 mg three times × day × 28 days)	1 after treatment: asymptomatic
Case 3	M	64	2	None	Liposomial Amphotericin B 3 mg/kg/day at day 1–5, 10 and 21	1 after treatment: asymptomatic
Case 4	M	53	2	None	Liposomial-Amphotericin B3 mg/kg/day at day 1–5, 10 and 21	2 after treatment: asymptomatic
Case 5	M	49	2	Obesity; recurrent tonsillitis episodes, especially during the winter months.	Liposomial-Amphotericin B3 mg/kg/day at day 1–5, 10 and 21	4 after treatment: asymptomatic

**Fig. 1:**
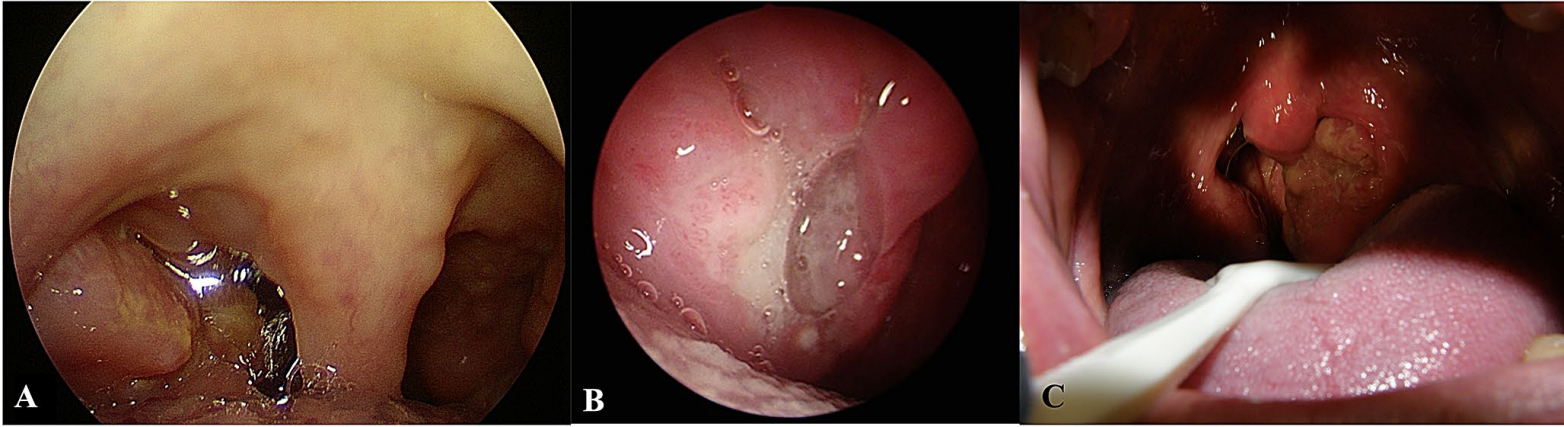
Different transoral presentation of oropharyngeal leishmaniasis. (**A**) Enlargement of the right tonsil with an irregular appearance, extending towards the base of tongue. (**B**) Tonsillar hypertrophy with central ulceration surrounded by granulation tissue. (**C**) Right tonsillar enlargement with a wide ulceration covered by fibrin (case 5)

**Fig. 2:**
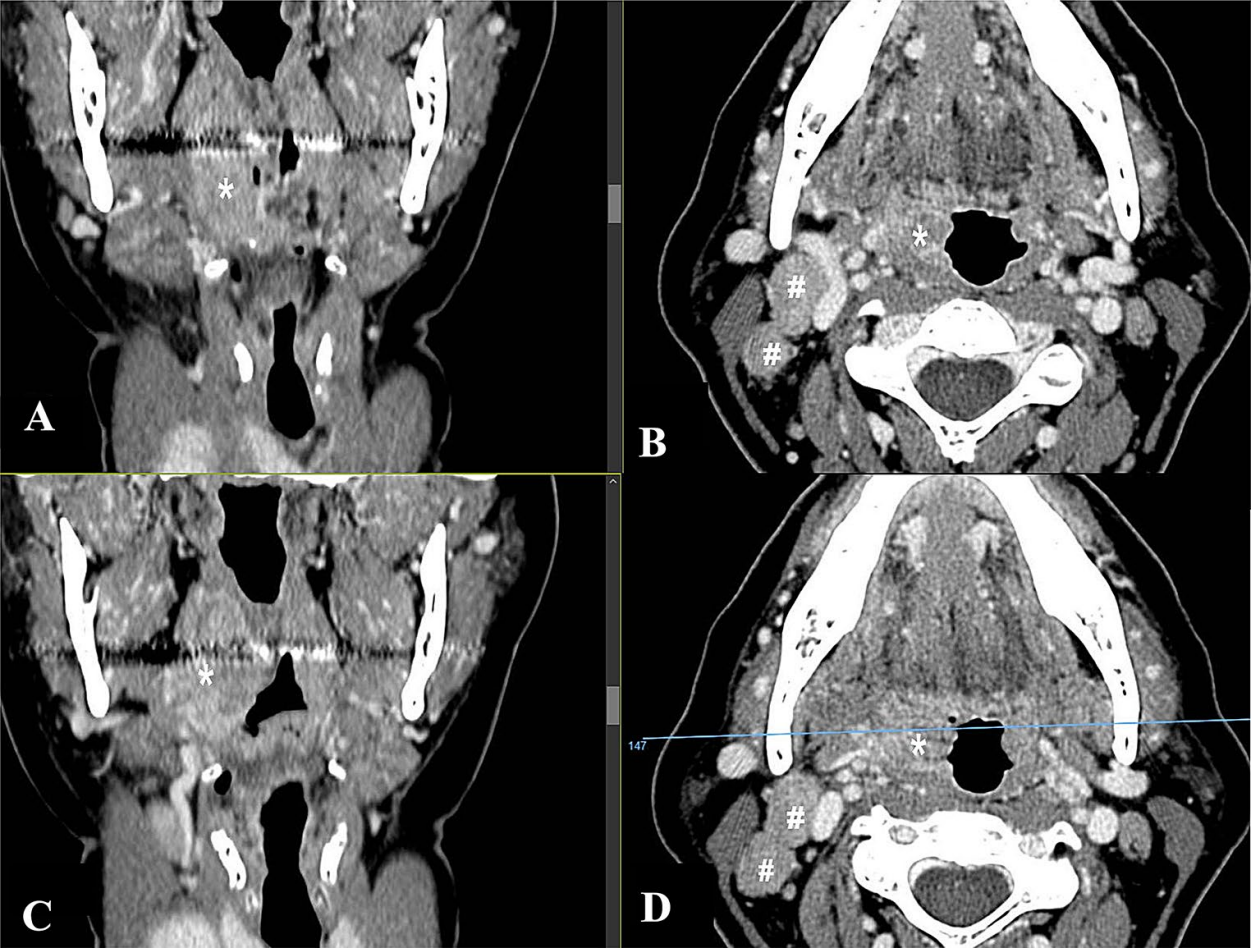
**A-B-C-D** Radiological images (case 1). (**A**-**C**) CT images with contrast enhancement coronal views and (**B**-**D**) axial views. The star (*) indicates the right palatine tonsil, where pathological infiltration of the right tonsillar fossa is evident. The infiltration extends downward along the palatoglossal fold and tapers off toward the ipsilateral glossoepiglottic vallecula. The hash symbol (#) highlights multiple pathological lymph nodes at levels II and III on the right side, with the largest node presenting an axial diameter of 2 cm

Five patients were included in the present study, four males and one female, with an age range between 42 and 64. All patients presented with unilateral pharyngodynia. Symptoms had started one (in 2 cases) or two (in 3 cases) months earlier. In all cases, transoral examination showed unilateral tonsillar swelling, with ulcerations in the surface, associated with granulated mucosa and covered with fibrin, being suspicious for malignancy (Fig. 1A-B-C). In two cases (case 1 and case 5), the CT scan demonstrated homolateral neck lymph node enlargement, while case 3 presented bilateral lymphadenopathy. Furthermore, in case 1, the enlargement of the palatine tonsil extended downward along the palatoglossal fold, tapering towards the glossoepiglottic vallecula on the same side. (Fig. 2A-B-C-D). In case 5, splenomegaly was detected via ultrasound, further increasing the suspicion of malignant neoplasms. No additional symptoms were referred. The medical history for case 1 was significant for type II diabetes, controlled by metformin therapy, while case 5 was significant for obesity. No comorbidities were registered in the remaining three cases. In all cases a biopsy was performed for diagnostic purposes. Tuberculosis and sarcoidosis were excluded by radiological and laboratory tests, including chest X-ray and QuantiFERON ^®^TB-Gold (QFT-G) test, and angiotensin-converting enzyme (ACE), respectively.

### Histopathological Findings

**Fig. 3:**
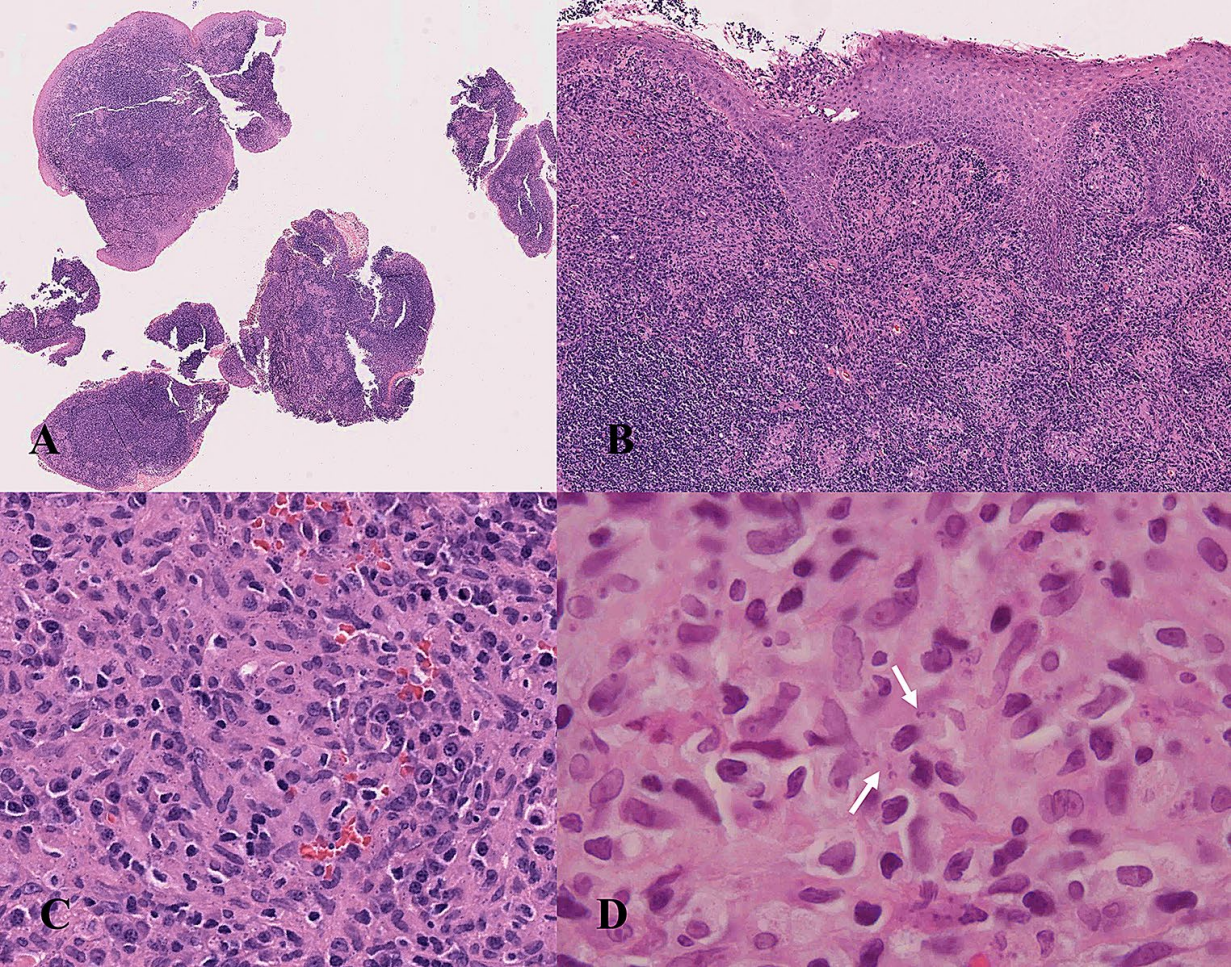
**A-B-C-D** Histological images of a tonsillar biopsy (case 4). (**A**) Biopsy fragments prepared for diagnosis (original magnification 1x). (**B**) The tonsillar squamous epithelium showed significant hyperplastic and regenerative changes with foci of erosive inflammation, while the subepithelial connective tissue exhibits a granulomatous inflammatory infiltrate with multinucleated giant cells, without necrosis (original magnification 10x). (**C**) Numerous amastigotes are evident in the cytoplasm of histiocytic cells (original magnification 40x) that appear as round to oval basophilic intracytoplasmic inclusions, approximately 4 μm in size. In (**D**) arrow indicates an amastigote with identifiable kinetoplast (original magnification 100x)

**Fig. 4:**
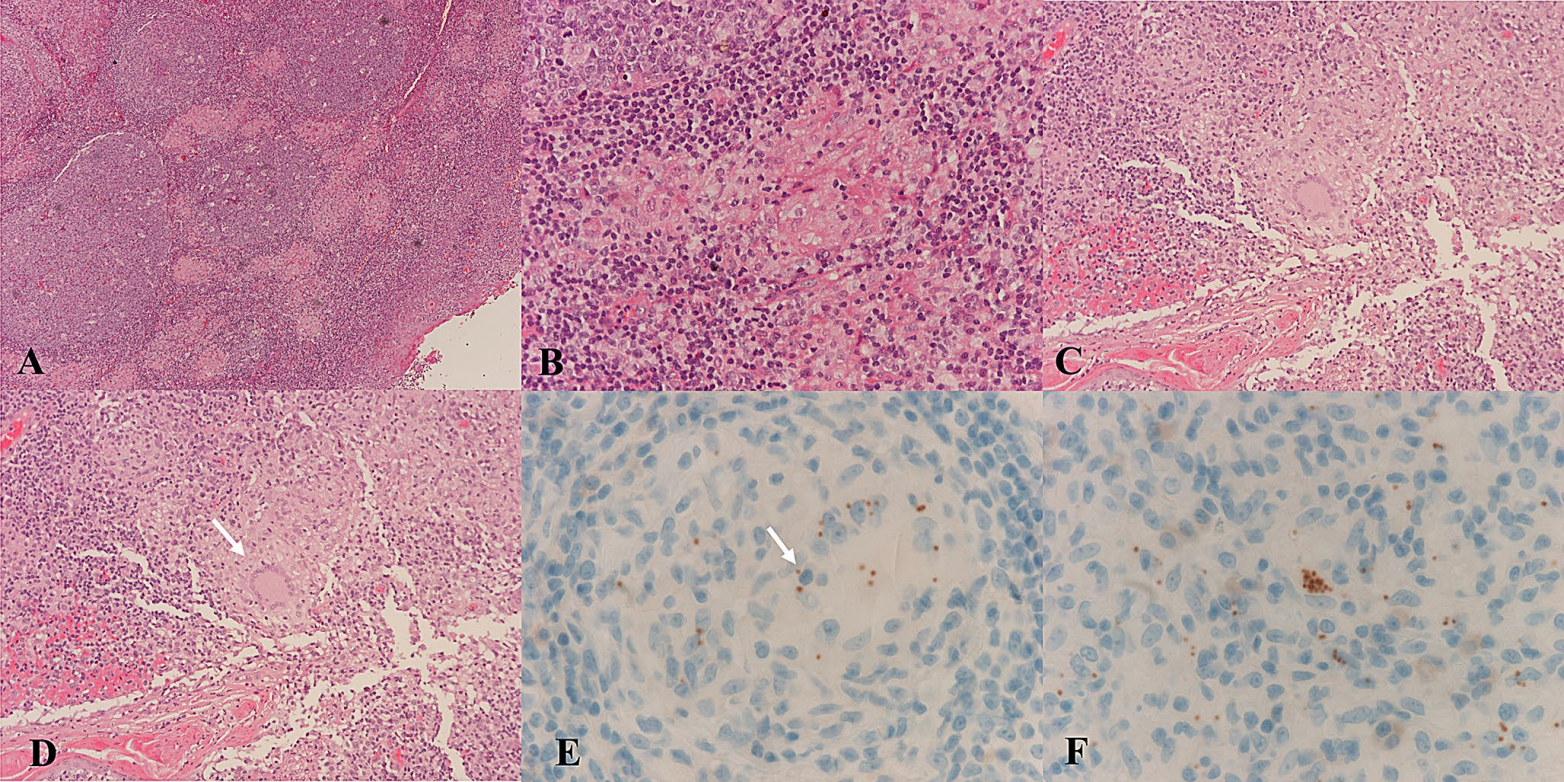
**A–E** Histological and immunohistochemical images of a tonsillar biopsy (case 1). (**A**) The intense chronic inflammatory reaction in the tonsil tissue is already evident at low magnification (original magnification 5x). (**B**) The inflammatory infiltrate is characterized by non-necrotizing granulomas (original magnification 20x). (**C**) The non-necrotizing granulomas are characterized by histiocytes and multinucleated giant cells (original magnification 10x). (**D**) A characteristic multinucleated giant cell, Langerhans type (original magnification 20x). (**E**) Immunohistochemical positivity for CD1a highlights intracellular amastigotes (original magnification 40x), which may also appear in clusters (**F**)

All the biopsies presented multiple non-necrotizing giant cell granulomas. Acid alcohol fast bacilli and fungi were searched with negative results. In three cases (case 3, 4 and case 5) amastigotes were detected on H&E (Fig. 3A-B-C-D). Giemsa and CD1a helped to reveal amastigotes in three out of five cases (Fig. 4A-B-C-D-E-F). In case 1 no parasites were detected via histological examination and the biopsy was repeated twice because the suspicion of malignancy was very high. The surface epithelium presented hyperplastic regenerative changes, but no dysplasia was detected. No evidence of malignancy was detected on histological examination.

### Microbiological Findings

In all the cases leishmanial DNA was detected in FFPE sections, with amplification curve for both targets (i.e. kDNA and rDNA by real-time PCR). Therefore, the diagnosis of tonsillar leishmaniasis was confirmed through molecular methods in all 5 cases. In one case, where no parasites were detected via histological examination, PCR was the only test that yielded a positive result. In case 2 and 5, real-time PCR testing for Leishmania DNA in peripheral blood yielded negative results; however, antileishmanial IgG detection was positive. Furthermore, anti-*Leishmania* antibodies were detected by immunoenzymatic assay and rK39 immunochromatographic test (ICT) in case 2 and by indirect immunofluorescence assay as well as rK39 ICT in case 5, respectively.

### Treatment

**Fig. 5:**
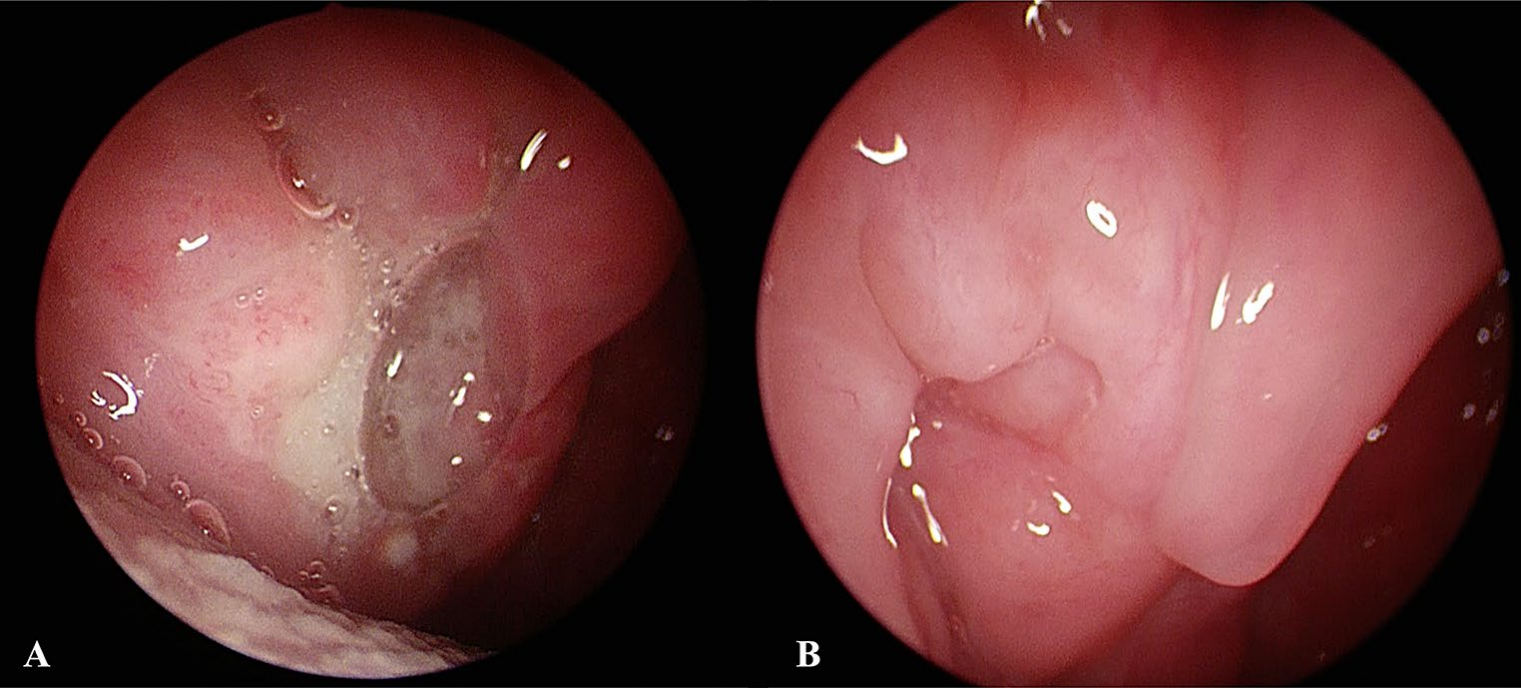
**A-B** A comparison of endoscopic transoral evaluation pre and post treatment (case 3). (**A**) the medial surface ulceration of the right enlarged palatine tonsil. (**B**) completely healed without any granulation tissue left

Case 1, 3, 4 and 5 were treated with liposomal Amphotericin B 3 mg/kg/day at day 1–5, 10 and 21. Case 2 was treated with Pentamidine isethionate (4 mg/kg, three infusions over 5 days), plus Miltefosine (50 mg three times × day × 28 days). In all 5 cases, complete remission of symptoms was achieved, particularly the pharyngodynia. Transoral clinical evaluation was performed in all cases and confirmed complete healing, with no evidence of tonsillar swelling (Fig. 5A-B).

## Discussion

TL is the most common form of leishmaniasis, with over 1 million new cases each year worldwide [17]. If not properly treated, the disease can leave life-long scars and cause serious disability or stigma.

Although not fatal, TL has a socioeconomic impact due to the stigmatization of individuals who may present disfiguring lesions when infected and even after being cured. *Leishmania braziliensis* is responsible for ML in Latin America [2]. A small percentage of persons infected with *L. braziliensis* develop mucous membrane involvement of the nose, oral cavity, pharynx, or larynx months to years after their skin lesions have healed [2–3]. Mucosal disease can occur because of infection due to other parasite species, including *L. tropica* (18–19), *L. donovani* [20–21–22–23–24] and *L. infantum* [3–[25-[26−[12-[16. *L.infantum*-associated ML appears to be more frequent than previously expected and manifests as localized mucosal disease in the absence of concomitant visceral or cutaneous leishmaniasis.

Tonsillar leishmaniasis can be detected in cases of multiple mucosal and/or visceral localization, nonetheless this series includes 5 cases of TL with exclusive localization in the tonsils. The exact mechanism of isolated mucosal/oropharyngeal disease without skin involvement remains unclear. Mucosal leishmaniasis is thought to arise not from a direct bite at the mucosal site, but rather as a metastatic consequence of an earlier, unnoticed cutaneous infection that subsequently spread to mucosal tissues [3–27].

The broad spectrum of ML clinical symptoms can complicate the process of obtaining an accurate diagnosis. Indeed, clinical manifestations of ML are variable and often nonspecific, including nodules, polypoid lesions or granular inflammation and may involve the buccal area, pharyngeal and laryngeal regions and, less frequently, the nose [28]. ML is often misdiagnosed with other upper respiratory tract diseases, particularly cancer, since they frequently present with ulcerated and bleeding masses. Due to the overlap in the clinical presentation of ML and neoplasia, leishmaniasis should be taken into consideration in the differential diagnosis of head and neck cancer, especially in patients from endemic areas or who report travelling to endemic countries [29].

On histology all cases presented multiple non-necrotizing granulomas. Tonsillar granulomatous lesions can have multiple aetiologies, among which infectious and non-infectious conditions should be considered. In the present cases, tuberculosis and sarcoidosis were excluded by clinical, radiological and laboratory tests. In 4 out of 5 cases amastigotes were detected either on H&E only or with the CD1a immunostaining, while in 1 case TL was suspected considering the recent increase of TL cases in northeastern Italy [1].

Other differential diagnoses to consider for parasitized histiocytes in the oropharynx, besides tuberculosis, include histoplasmosis, cryptococcosis, and talaromycosis. All these infectious diseases are caused by pathogens that can trigger a granulomatous immune response, which varies from being absent in immunocompromised individuals to forming large necrotizing lesions in people with an immunocompetent immune system. The diagnosis of TL is based on the precise identification of the causative pathogen [17]. For this purpose, conventional methods such as microscopic examination of samples stained with hematoxylin-eosin (H&E), Giemsa, Grocott and periodic acid–Schiff (PAS), specific cultures, and PCR amplification of *Leishmania* DNA are used. Amastigotes appear as round to oval basophilic intracytoplasmic inclusions, approximately 4 μm in size, found in varying numbers within the cytoplasm of histiocytes [30]. They contain a single, dense, eccentric nucleus and a rod-shaped kinetoplast, which can be oriented either perpendicular or parallel to the nucleus. Kinetoplasts can be challenging to observe in fixed sections of Leishmania-infected tissue, regardless of the staining technique used. This difficulty is especially pronounced in sections stained with hematoxylin and eosin. Other pathogens that must be microscopically differentiated include: *Histoplasma capsulatum*, *Cryptococcus neoformans and Talaromyces marneffei* [31]. These pathogens are approximately the same size and primarily found within macrophages and histiocytes. Moreover *Histoplasma capsulatum* and *Cryptococcus neoformans* infections may occasionally occur in Italy (32–33), complicating the interpretation of epidemiological data.

*Histoplasma capsulatum* causes histoplasmosis, a systemic fungal infection affecting humans and animals, often linked to HIV. Since the 1980s, it has become a significant opportunistic infection in AIDS patients. *Histoplasma capsulatum* appears in round or ellipsoidal forms, and the cell membrane is not distinctly visible. A purplish nucleus, occupying about one-third to one-half of the spore and the cytoplasm appeared light blue. Peripheral spores are seen as empty, bright, colorless rings, resembling a capsule, a sort of halo-like ring around the cell, and display narrow-based budding, often forming clusters of numerous organisms and they are strongly positive for PAS, while amastigote forms of *Leishmania spp* are not stained. The distinctive kinetoplasts of *Leishmania*, which generally stain more intensely than the nucleus, are not present in *Histoplasma capsulatum*.

*Cryptococcus* is a yeast with a protective capsule, commonly found in the environment. It is a globally distributed opportunistic fungal pathogen, and it has been isolated from sources such as pigeon droppings, decomposing wood, fruits, and vegetables. Individuals with weakened cell-mediated immunity, including those with HIV, organ transplant recipients, and patients on long-term corticosteroid therapy, are at the highest risk of developing cryptococcal infections. *Cryptococcus neoformans* is typically identified in tissue sections as a round yeast, both intracellular and extracellular, ranging from 5 to 10 μm in size with some variation. It possesses a thick polysaccharide capsule, which creates a clear halo-like space in H-E staining and shows positivity with Alcian blue staining. Although histopathological criteria can often help distinguish cryptococcosis from histoplasmosis, differentiating between these infections can sometimes be challenging, and their treatments differ. However, the presence of yeasts with indentations or irregular depressions in their cell walls may be more indicative of cryptococcosis rather than histoplasmosis [34].

Talaromycosis is a serious fungal infection caused by *Talaromyces marneffei*, formerly *Penicillium marneffei*, a thermally dimorphic fungus and major pulmonary pathogen. It can spread through the lymphatic system or bloodstream, causing life-threatening complications. More common in warm, humid climates, it is prevalent in Southeast Asia, northern India, and southern China, affecting individuals with travel history to these regions [35]. *T. marneffei* pathogens are primarily found within the cytoplasm of macrophages, with a few scattered extracellularly. The yeast forms of *T. marneffei* appears round, ellipsoidal, or sausage-shaped, featuring one or two small purplish-red nuclei and a light blue cytoplasm. The pathogens varied in size, ranging from 2 to 8 μm, and their cell walls were not distinctly visible. In PAS staining, however, *T. marneffei* cell walls appeared red, well-defined, and continuous [36]. As our patients did not report travels to Asia, we did not consider it as a potential differential diagnosis.

Althoughthe distinction on the H-E-stained slide may be difficult, PAS and Grocott-stained slide could help to solve this diagnostic issue (positive stain in *H. capsulatum*, in Cryptococcus neoformans and in Talaromyces marneffei and negative stain in Leishmania spp.).

However, in more advanced lesions, a dense tuberculoid or sarcoid granulomatous infiltrate may be observed, along with a reduction in the parasitic load. In such cases, skin or mucosal biopsies might reveal very few or even an almost complete absence of amastigotes, making additional techniques necessary to highlight the parasite’s presence [37]. In these cases, immunohistochemistry using anti-CD1a or anti-*Leishmania* antibodies has shown greater sensitivity for detecting amastigotes and a valuable tool that can facilitate pathogen detection (38–39).

Additionally, the detection of *Leishmania* parasites DNA via PCR offers even greater sensitivity, ranging from 75.7 to 100%, while maintaining a specificity of 100%. Both histological and molecular tools demonstrate high diagnostic accuracy [40]. In our cohort of patients, in 4 out of 5 cases amastigotes were detected either on H&E only or with CD1a immunostaining, while in 1 case ML was suspected considering the recent increase in TL cases in northeastern Italy and was subsequently confirmed by PCR.

Most previous studies utilized the CD1a antibody from Novocastra/Leica Biosystems, derived from the MTB1 clone. Gadelha et al. conducted a comparison between the MTB1 and O10 clones, demonstrating that the MTB1 clone provided greater sensitivity, specificity, and accuracy [41]. In contrast, Lopez-Trujillo et al. used the routine CD1a (EP3622) Rabbit Monoclonal Antibody from Ventana-Roche Tissue Diagnostics, which showed a homogeneous staining pattern, often with greater intensity at one pole [40]. In this study (in 3 out of 5 cases), the CD1a (EP3622) Rabbit Monoclonal Antibody from Ventana-Roche Tissue Diagnostics was used. It is also important to highlight that from a histological perspective, in all analyzed cases, the squamous epithelium showed significant hyperplastic and regenerative changes, but there were no morphological features indicative of dysplasia and/or neoplasia.

According to the WHO, the treatment of ML varies between countries, considering the infecting species. Current treatments offer varied efficacy rate and diverse systemic effects that often lead to the patient abandoning treatment. In each case therapy is tailored according to clinical presentation, infecting *Leishmania* species and immunological status of the patient [42]. Patients must undergo to systemic treatment prevent morbidity, as disfigurement, and mortality related to aspiration pneumonia and/or respiratory obstruction [29].

The treatment strategy used in the current cases mirrored the successful therapeutic approach adopted in three previous ML cases [29]. All patients reached a complete remission after systemic treatment. At present, there is no standardized treatment for ML. In each case therapy is tailored according to clinical presentation, infecting *Leishmania* species and immunological status of the patient [43]. Drugs with distinct activity against Leishmania, including liposomal amphotericin B and pentamidine were used. Amphotericin B (AmB). AmB can accumulate on membrane surfaces, disrupting *Leishmania* cells by extracting crucial lipids, ultimately causing cell death. This interaction is significantly stronger than its binding to cholesterol in human cells, emphasizing its selective affinity, which is essential for its mechanism of action. The main limitation of AmB is its poor water solubility, which led to the use of its nephrotoxic deoxycholate formulation as a second-line therapy for VL, CL, and MCL since the 1960s. The introduction of liposomal delivery systems in the 1970s enabled the development of AmBisome, a liposomal formulation designed to enhance bioavailability and minimize toxicity [44]. Pentamidine, a synthetic amidine derivative became a treatment option for drug-resistant CL in the 1970s [45]. Pentamidine interacts with nucleic acids, interfering with nucleotide incorporation and oxidative phosphorylation, which in turn disrupts the synthesis of DNA, RNA, phospholipids, and proteins. Additionally, this drug may bind to kinetoplast DNA, inhibiting mitochondrial respiratory chain complex II and triggering apoptosis by increasing intracellular calcium levels [44].

Combined dermatological and otolaryngologist follow-up after treatment is important for the clinical management of ML. A biopsy of the lesion is mandatory to perform a parasitological diagnosis, thus allowing the prompt introduction of a correct antileishmanial therapy.

## Conclusions

The oropharynx is an anatomical region rich in lymphoid tissue and features the unique reticulated tonsillar crypt epithelium, making it a significant site for virus-related carcinogenesis in the head and neck. Considering the similarities in clinical presentation of isolated ML and tumours, tonsillar leishmaniasis should be taken into consideration in the differential diagnosis of head and neck cancer, especially in patients from - or who report travelling to - endemic areas.

## Electronic Supplementary Material

Below is the link to the electronic supplementary material.

Supplementary Material 1: Figure 1A-B: Post-treatment endoscopic follow-up (case 1). (A) Perfect symmetry of the tonsils is observed. (B) Disappearance of the hypertrophic/infiltrative appearance of the right tonsil and the base of the tongue.

Supplementary Material 2: Figure 2 A-B: Radiological images of case 2. The axial plane of the CT scan shows a slight volumetric increase compared to the contralateral side, with mild enhancement of the surface mucosa.

Supplementary Material 3: Figure 3 A-B-C: Radiological and histological image of case 3. (A) The axial plane of the CT scan points out a contrast-enhanced ulceration of the right palatine tonsil (black arrow) with associated bilateral reactive lymphadenopathies at the IIa level (*). (B) The inflammatory infiltrate is characterized by non-necrotizing granulomas (original magnification 10x). (C) Lympho-histiocytic infiltrate with scattered intracytoplasmic amastigotes (20x).

Supplementary Material 4: Figure 4 A-B-C-D: Histological images of case 5. (A) The intense chronic inflammatory reaction in the tonsil tissue is already evident at low magnification and it is characterized by non-necrotizing granulomas (original magnification 10x). (B) Non-necrotizing granulomas (original magnification 20x). (C) Lympho-histiocytic infiltrate with intracytoplasmic amastigotes (arrow) (40x). (D) Amastigotes are also highlighted by Giemsa histochemical staining (arrow) (40x).

## Data Availability

No datasets were generated or analysed during the current study.

## Abbreviations

VL: Visceral Leishmaniasis
CL: Cutaneous Leishmaniasis
ML: Mucosal Leishmaniasis
MCL: Mucocutaneous Leishmaniasis
TL: Tegumentary Leishmaniasis
DL: Diffuse Leishmaniasis
FFPE: Formalin Fixed Paraffin Embedded
ERR: Emilia-Romagna Region
PAS: Periodic Acid–Schiff
H&E: Hematoxylin-Eosin
AmB: Amphotericin B
