# Supplementary figures and images for: Tonsillar Leishmaniasis: A Rare Clinical Entity Mimicking Malignancy in the Oropharynx – A Case Series from Northeastern Italy
Source: Head Neck Pathol. 2025 Mar 26;19(1):39. doi: 10.1007/s12105-025-01773-3 (PMC11947358; doi:10.1007/s12105-025-01773-3)

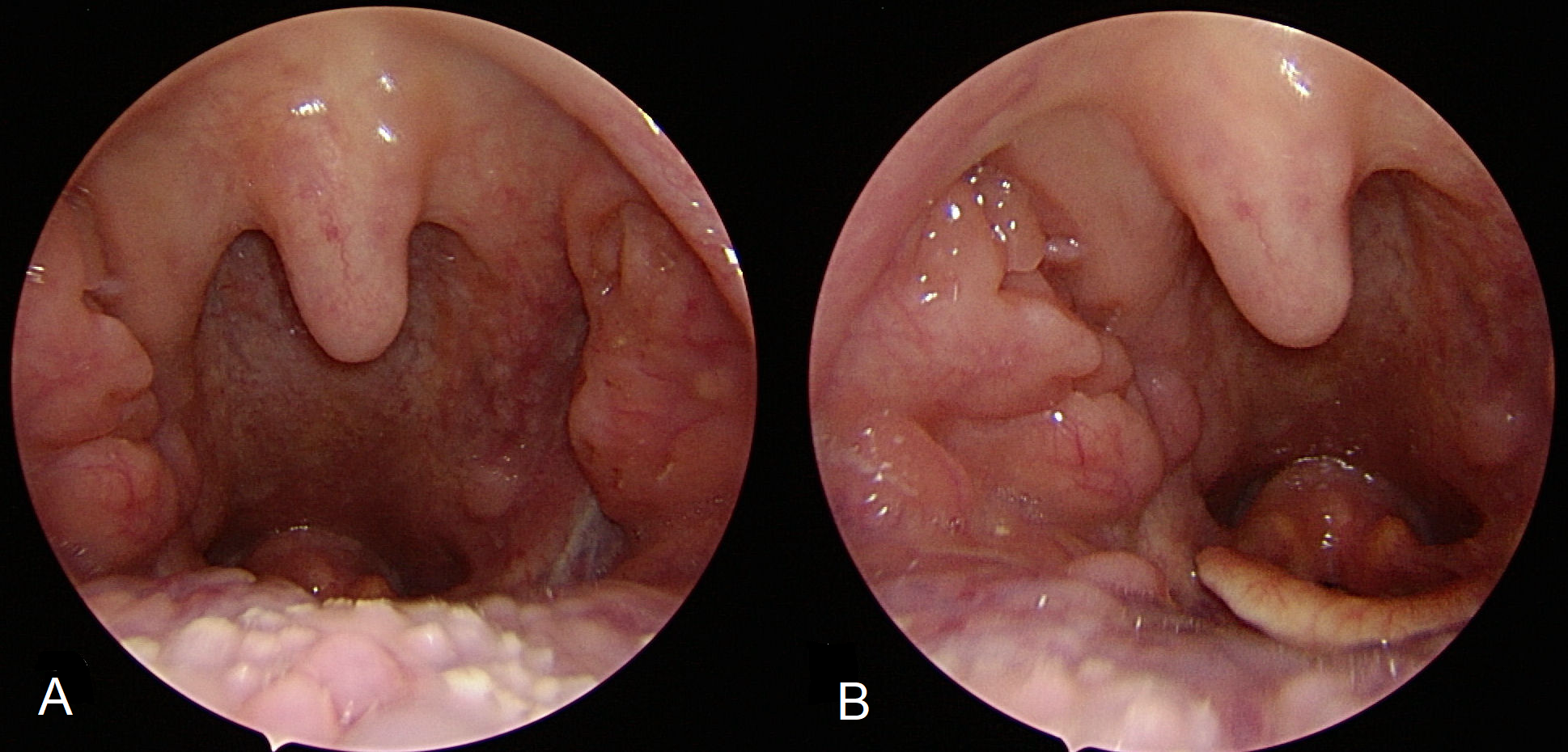

Supplement: Supplementary file 1 — Supplementary Material 1: Figure 1A-B: Post-treatment endoscopic follow-up (case 1). (A) Perfect symmetry of the tonsils is observed. (B) Disappearance of the hypertrophic/infiltrative appearance of the right tonsil and the base of the tongue. [file 12105_2025_1773_MOESM1_ESM.tif]

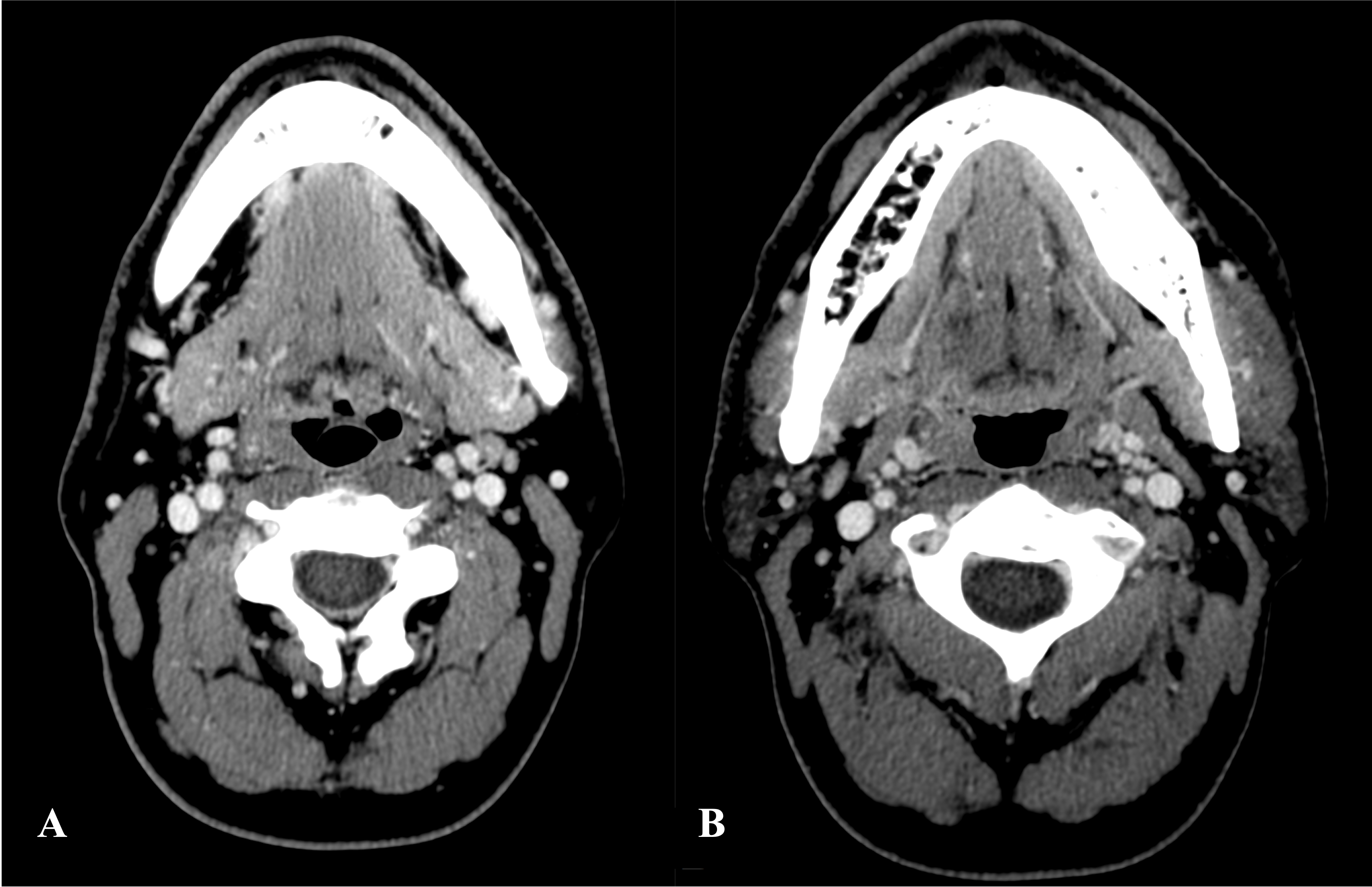

Supplement: Supplementary file 2 — Supplementary Material 2: Figure 2 A-B: Radiological images of case 2. The axial plane of the CT scan shows a slight volumetric increase compared to the contralateral side, with mild enhancement of the surface mucosa. [file 12105_2025_1773_MOESM2_ESM.tiff]

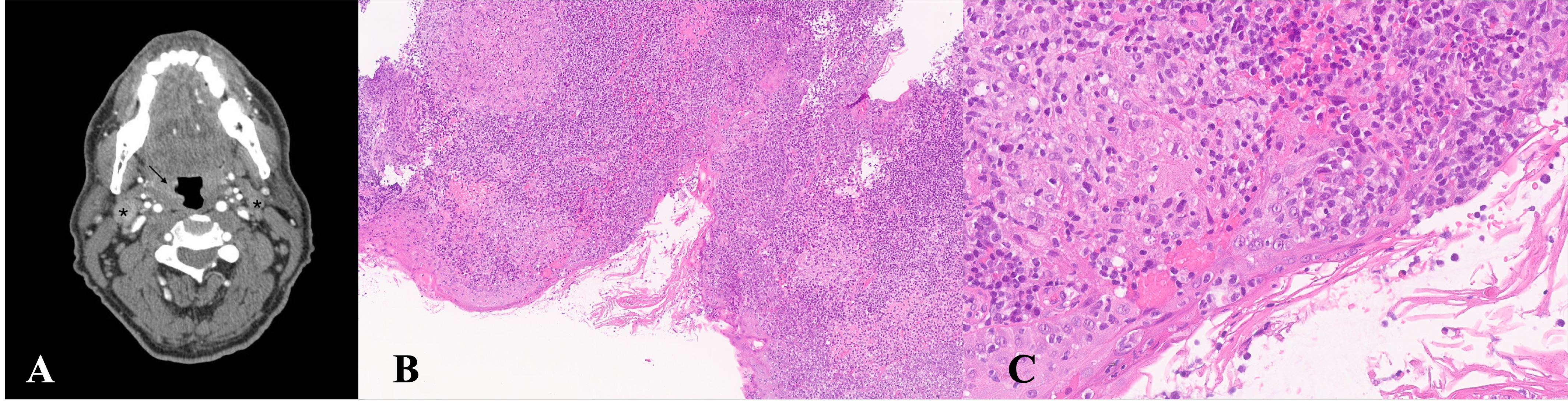

Supplement: Supplementary file 3 — Supplementary Material 3: Figure 3 A-B-C: Radiological and histological image of case 3. (A) The axial plane of the CT scan points out a contrast-enhanced ulceration of the right palatine tonsil (black arrow) with associated bilateral reactive lymphadenopathies at the IIa level (*). (B) The inflammatory infiltrate is characterized by non-necrotizing granulomas (original magnification 10x). (C) Lympho-histiocytic infiltrate with scattered intracytoplasmic amastigotes (20x). [file 12105_2025_1773_MOESM3_ESM.tiff]

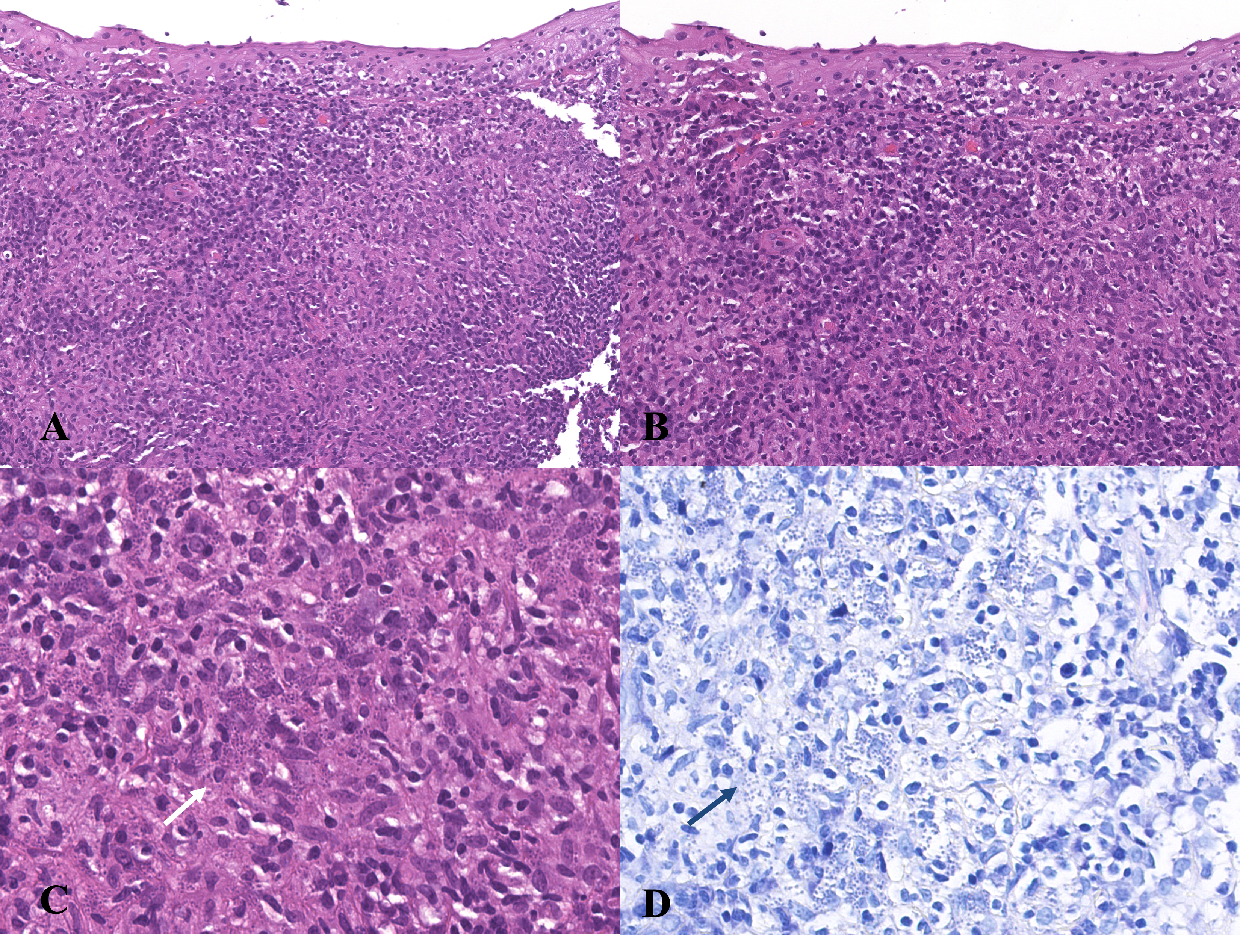

Supplement: Supplementary file 4 — Supplementary Material 4: Figure 4 A-B-C-D: Histological images of case 5. (A) The intense chronic inflammatory reaction in the tonsil tissue is already evident at low magnification and it is characterized by non-necrotizing granulomas (original magnification 10x). (B) Non-necrotizing granulomas (original magnification 20x). (C) Lympho-histiocytic infiltrate with intracytoplasmic amastigotes (arrow) (40x). (D) Amastigotes are also highlighted by Giemsa histochemical staining (arrow) (40x). [file 12105_2025_1773_MOESM4_ESM.tiff]
